# Increasing access to health workers in rural and remote areas: what do stakeholders’ value and find feasible and acceptable?

**DOI:** 10.1186/s12960-020-00519-2

**Published:** 2020-10-16

**Authors:** Onyema Ajuebor, Mathieu Boniol, Michelle McIsaac, Chukwuemeka Onyedike, Elie A. Akl

**Affiliations:** 1 Health Workforce Department, World Health Organization, 20 Avenue Appia, 1211, Geneva 27, Switzerland; 2 Department of Internal Medicine, American University of Beirut, Beirut, Lebanon

**Keywords:** Health workers, Rural areas, Guidelines

## Abstract

**Background:**

The primary aim of this study is to assess stakeholders’ views of the acceptability and feasibility of policy options and outcome indicators presented in the 2010 World Health Organization (WHO) global policy recommendations on increasing access to health workers in remote and rural areas through improved retention.

**Methods:**

A survey on the acceptability, feasibility of recruitment and retention policy options, and the importance of their outcome indicators was developed. It followed a cross-sectional approach targeting health workers in rural and remote settings as well as policy- and decision-makers involved in the development of recruitment and retention policies for such areas. Respondents were asked their perception of the importance of the policy outcomes of interest, as well as the acceptability and feasibility of the 2010 WHO guidelines’ policy options using a 9-point Likert scale.

**Results:**

In total, 336 participants completed the survey. Almost a third worked in government; most participants worked in community settings and were involved in the administration and management of rural health workers. Almost all 19 outcomes of interests assessed were valued as important or critical. For the 16 guideline policy options, most were perceived to be "definitely acceptable" and "definitely feasible", although the policy options were generally considered to be more acceptable than feasible.

**Conclusion:**

The findings of this study provide insight into the revision and update of the 2010 WHO guideline on increasing access to health workers in remote and rural areas. Stakeholders’ views of the acceptability, feasibility of policy options and the importance of outcomes of interest are important for the development of relevant and effective policies to improve access to health workers in rural and remote areas.

## Background

The availability of competent health workers is essential to the proper functioning of health systems [1, 2]. The World Health Organization (WHO) has estimated that the world will be short of 18 million health workers to deliver universal health coverage by 2030 [3]. These shortages are typically the most severe in rural areas, where almost half of the world’s population live [4]. With the objective of leaving no one behind and to progress toward universal health coverage (UHC), improving the attractiveness of rural area jobs to health workers is critical. Over decades, efforts have been made to attract health workers to rural, remote, and medically underserved areas. However, the success of these efforts has been limited due to barriers such as poor remuneration and health worker motivation [5, 6].

Recognizing the extent of the challenge, and in response to a request by Member States, WHO developed in 2010 the first edition of the global policy recommendations on increasing access to health workers in remote and rural areas through improved retention [7]. With the persistent challenges to retaining human resources for health (HRH) in rural and medically underserved areas, it became necessary to provide countries with more relevant and up-to-date guidance to support their efforts to address this challenge. In developing health systems guidelines, the views of stakeholders are important to consider as implementation of policy recommendations is more likely to succeed if the policy options are considered acceptable and feasible by stakeholders [8].

Much still needs to be understood about how stakeholders value approaches to recruit and retain health workers in rural areas and the impact of recruitment policies on outcomes [9–12]. This paper aims to get us a step further in redressing this knowledge gap by understanding how stakeholders’ values of the outcomes of interest and the acceptability and feasibility of policies to address rural and remote health worker recruitment and retention may affect the success of chosen policy interventions. In providing this insight, the study also aims to inform the revision of the global policy recommendations on increasing access to health workers in remote and rural areas through improved retention.

## Materials and methods

The survey followed a mixed method cross-sectional design comprising of quantitative and qualitative aspects. We collected the data between 25 September 2019 and 31 December 2019.

### Survey population

For the purpose of this study, we defined key stakeholders as people involved in the policy formulation, administration, or management of health workers serving in rural areas. These stakeholders could be health workers themselves, or decision-makers appointed by governments and authorities to manage health worker service provision in rural and underserved areas. Respondents can also be clinical practitioners or health service managers or act in both capacities; they could also be based in rural areas, or in urban areas but working on rural health worker policies or administration. Participation in the survey was voluntary, responders consented to the survey, and all responses were anonymous. Ethical clearance was obtained from the WHO Ethics Review Committee – ERC0003207. In keeping with the WHO standards, at least two independent reviewers representing public interests were invited to review the study protocol for methodological and ethical considerations as part of the ethics review clearance process.

Based on a previous survey conducted for community health workers [13], a minimum sample size for this survey was calculated to enable the identification of a difference of 1.5 in the Likert scale score (ranging from 1 to 9), in detecting any relationships between one category of a respondent's factor, such as the WHO region they represent, and others such as their gender and occupation. Because the Likert scale were expected to be non-normally distributed, the response pattern was set to an average score of 7 as in the acceptability and feasibility of policies for the community health workers survey mentioned earlier [13]. A minimum sample of 200 respondents, factoring a 10% non-response rate was then applied after considering these inputs. This minimum sample size also enables a precision level of less than 0.5 of the average scores in the acceptability and feasibility Likert scales. (95% confidence interval of the mean).

### Survey questionnaire

The design of the questionnaire (see additional file 1) followed that of similar questionnaires developed for previous WHO guidelines on rehabilitation services [14] and community health workers [13]. Accordingly, three broad areas were considered for assessment and discussion:

- Values assigned to outcomes of the guideline recommendations
- Acceptability of the policy options being recommended
- Feasibility of the policy options being recommended

Respondents were asked to rank the importance of the outcomes of interest on a 9-point Likert scale. The outcomes scale had three anchor points: *Not important* (1), *Important* (5) and *Critical* (9). The Likert scale to rank participants’ perception of the acceptability and feasibility of the policy options was also anchored along similar points: *Not acceptable* (1), *Uncertain whether acceptable or not* (5) and *Definitely acceptable* (9), and *Not feasible* (1), *Uncertain whether feasible or not* (5) and *Definitely feasible* (9). Binary answers (‘Yes’ or ‘No’) were used to capture the need for guidance on the four categories of policy options (education, regulation, finance, and professional support). In addition, stakeholders were asked to rank a set of five common barriers to implementing such policies (tools/infrastructure, experts’ technical support, financial incentives, government legislation/policies and community dynamics) on a scale of 1 through 5—the score of 1 being for the least important per category and the score of 5 pegged to the most important. In order to reach all WHO regions, questionnaires were made available in the six WHO official languages English, French, Spanish, Chinese, Russian and Arabic languages.

### Data collection process

The questionnaire was pilot-tested before dissemination. The collection of responses was conducted with a subscription version of Survey Monkey. To reach the desired stakeholders, the links to survey were disseminated through multiple channels in line with the sampling strategy, including the World Organization of Family Doctors (WONCA) rural health expert database, Health Information for All (HIFA) online community, WHO Global Health Workforce Network (GHWN) and the WHO health workforce and regional offices websites and newsletter distribution outlets.

### Data analysis

The responses to the 9-point scale factors were reported as mean, median and interquartile range to account for the non-normal distribution of the observations. Visual inspection of the distribution of responses was also conducted to identify potential outliers. Because of non-normality, the percentage of respondents giving the highest possible score (i.e. 9) was also reported. For all outcomes and policy options where the highest score was chosen by at least 40% of the respondents, a comparison analysis of giving a score of 9 versus less was conducted with Fisher test across regions, occupations and gender. The average difference between the acceptability score and feasibility score was computed and compared to zero on a *t*-test to identify policy options more acceptable than feasible and vice versa. All tests were two-sided and a *P* value of < 0.05 was considered as statistically significant. Because of the explorative nature of the study, no attempt to correct for multiple testing was done. Analyses were conducted using the Stata software version 14.2. Comments received through open-ended questions were presented as narrative summary.

## Results

### Demographic and occupational characteristics of respondents

A total of 336 respondents completed the survey. Demographic and occupational characteristics of the respondents are presented in Tables 1 and 2 respectively.

**Table 1:** Demographic characteristics of respondents

	*N*	%
*Region*		
Africa	55	16
South and Southeast Asia	71	21
Americas	34	10
Europe	102	30
Eastern Mediterranean	6	2
Western Pacific	18	5
Missing values	50	15
*Gender*		
Female	180	53.6
Male	133	39.6
Other	1	0.3
Missing values	22	6.5
*Age*		
< 25	4	1
25–39	144	42
40–54	106	32
55–64	49	15
65+	15	4
Missing values	18	5
*Educational qualification*		
Master’s degree and above	279	83
Other degrees/certificates	36	11
Missing values	21	6

**Table 2:** Occupational characteristics of respondents

	*N*	%
*Occupation*		
Physicians	183	54
Dentists	29	9
Academics	28	8
Policy makers/health service managers	22	7
Nursing and midwifery personnel	17	5
Other associates	10	3
Other professionals	6	2
Missing values	41	12
*Challenged by attraction, recruitment and retention of health workers in country of work*		
Yes	309	92
No	27	8
*Based in a rural location*		
Yes	211	63
No	125	37
*Involved in the management or administration of health workers*		
Yes	198	59
No	138	41
*Involvement in rural or remote policy programmes*		
Not applicable	148	44
Influenced policies	115	34
Developed policies	73	22

**Fig. 1:**
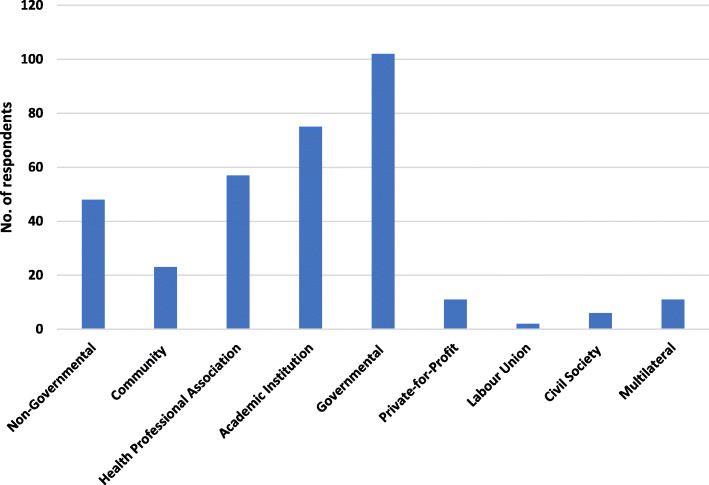
Main organization of respondents. This figure shows the number of respondents according to the type of organization for which they work. Multiple choices were allowed for this question

**Fig. 2:**
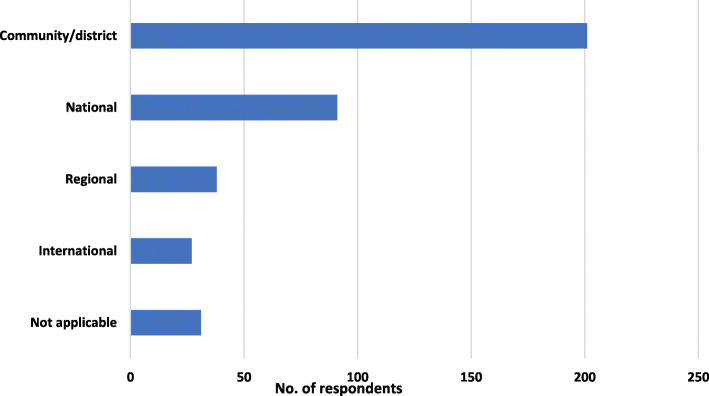
Geographical scope of work. This figure shows the number of respondents according to the geographical scope in which they work. Multiple choices were allowed for this question

All geographical regions are accounted for with a high representation from Europe and Southeast Asia. In total, 90% of the respondents fell within an active workforce age range (between 25 and 64). There was reasonable gender balance among the respondents with slightly more representation from females. Almost a third of the respondents worked in government; most worked at a local or community level and were involved in the administration and management of rural health workers (see Figs. 1 and 2). More than half of the respondents were also involved in influencing or developing policies for rural health workers.

### Outcome valuation

Across all regions, the highest-rated outcome (based on the percentage respondents scoring 9) was *improved rural attractiveness to health workers* (52%); this rating was highest for European respondents compared to those from other regions (66%, *P* = 0.025). The second highest-rated outcome was *improved availability of health workers* (45% rating it at 9); this showed minor regional variation with lowest rating observed for European respondents (33%, *P* = 0.092). The third highest-rated outcome was *improved motivation (*42% rating it at 9); this outcome was scored higher for respondents representing the African region (58%, *P* = 0.01). The fourth highest-rated outcome was *improved rural recruitment* (40% rating it at 9). Regional variations were observed (with rating at 9) for 35%, 55% and 71% for respondents from Europe, the Americas and Western Pacific respectively (*P* = 0.041). Table 3 presents the summary statistics of participants’ ratings of the different outcomes of interest.

**Table 3:** Values attached to outcome of interest; answers provided on a 9-point Likert scale

*Outcome*	*Mean*	*% with a ‘9’ rating*
*Workforce performance*		
Improved availability of health workers	7.5	45%
Improved competence of health workers	7.3	37%
Improved responsiveness of health workers to community needs	7.2	31%
Improved productivity of health workers to do tasks more efficiently	6.8	22%
Improved public/community recognition of rural/remote health workers	7.3	36%
Improved cooperation (between rural and urban health workers)	7.0	31%
Improved motivation	7.4	42%
Improved personnel development and lifelong learning opportunities	7.4	36%
Reduced turnover rate of health workers leaving remote/rural posts	7.3	39%
*Health systems*		
Improved accessibility (coverage of interventions)	7.3	36%
Improved quality of care	7.3	33%
Improved productivity (of the health system)	7.1	31%
Improved social accountability	7.0	27%
Improved responsiveness	7.0	25%
Improved practice environment (including supportive supervision)	7.4	34%
Improved rural recruitment of health workers	7.3	40%
Improved rural attractiveness to health workers	7.8	52%
Improved workforce skills mix	7.0	26%
Improved scope of practice	6.7	20%

When comparing the rating of physicians and non-physicians, there were no differences for *improved availability of health workers* and for *improved rural recruitment of health workers*. However, there were differences between physicians and non-physicians for the ratings of *improved motivation* with physicians rating this higher than non-physicians (48% versus 36%; *P* = 0.051) and of *rural attractiveness to health workers* (58% versus 43%; *P* = 0.017). No statistically significant differences were observed between males and females.

### Acceptability and feasibility findings of the policy options

All policy options were deemed to be acceptable though two received a lower rating compared to the others: *Location of health profession schools outside major cities* and *Impose a compulsory service in rural areas in exchange of licensing or other employment benefits*. The two policy options that received the highest scores of acceptability were *Improve living conditions for health workers and their families and invest in infrastructure and services in rural areas* and *Provide a safe and supportive working environment for rural and remote posts*.

For feasibility, most of the policy options were deemed to be feasible, even though the ratings were generally lower than the acceptability ratings across the policy options. Respondents found all but one policy option (*imposing compulsory service in rural areas*) to be statistically significantly more acceptable than feasible. Table 4 summarizes the perceived acceptability and feasibility of the policy options recommended in the 2010 guidelines while Table 5 summarizes the comparison of acceptability and feasibility score by region, occupation and gender. More detailed information on Tables 3, 4 and 5 are included in additional file 2.

**Table 4:** Stakeholders’ perception of the acceptability and feasibility of the guidelines policy options; answers provided on a 9-point Likert scale

	Acceptability		Feasibility	
	*Mean*	*% with a ‘9’ rating*	*Mean*	*% with a ‘9’ rating*
*Education*				
Targeted admissions of students from a rural background into health profession schools	7	37%	6.7	30%
Location of health profession schools outside major cities	6.4	30%	5.9	20%
Provide clinical rotations/community experiences in rural areas during pre-service education	7.8	51%	7.5	44%
Revise the curricula of pre-service education to include rural health issues, skills for team-building and supervision, and primary care orientation	7.9	50%	7.5	43%
Continuing education and professional development programmes that meets the needs of rural health workers	8	54%	7.7	43%
*Regulatory*				
Enhance the scope of practice of specific cadres of health workers in rural areas	7.6	38%	7.1	30%
Produce different types of health workers with appropriate training and regulation for rural practice	7.1	32%	6.7	27%
Impose a compulsory service in rural areas in exchange of licensing or other employment benefits	6.1	23%	6.1	24%
Scholarships or other type of financial incentives for education in exchange of return of service in rural or remote areas	7.7	50%	7.4	39%
*Financial incentives*				
Provide appropriate financial incentives (monetary or non-monetary)	7.9	52%	7.4	42%
*Professional and personal support*				
Improve living conditions for health workers and their families and invest in infrastructure and services in rural areas	8.2	64%	7.3	40%
Provide a safe and supportive working environment for rural and remote posts	8.3	66%	7.4	38%
Implement appropriate outreach support activities	8	53%	7.4	37%
Support career development programmes	8.1	56%	7.7	44%
Support the development of professional networks	7.8	48%	7.4	41%
Adopt public recognition measures	7.5	44%	7.3	39%

**Table 5:** Factors associated with level of acceptability and feasibility: by region, occupation and gender for four selected policy options

Policy options	Region	Occupation (physicians vs non-physicians)	Gender
*Acceptability*			
Continuing education and professional development	More acceptable in Americas and western pacific (76% and 80%) and less in Europe (49%), *P* = 0.04	No difference (*P* = 0.45)	No difference (*P* = 0.18)
Improving living condition, infrastructure and service in rural areas	No difference (*P* = 0.34)	No difference (*P* = 0.19)	No difference (*P* = 0.40)
Providing safe and supportive environment in rural and remote posts	No difference (*P* = 0.08)	No difference (*P* = 0.30)	More acceptable for women and men (72% vs 56%), *P* = 0.006
Support career development programmes	No difference (*P* = 0.74)	No difference (*P* = 0.25)	No difference (*P* = 0.12)
*Feasibility*			
Continuing education and professional development	More feasible in Americas and Africa (58% and 57%) and less in Southeast Asia (30%), *P* = 0.029	No difference (*P* = 0.45)	No difference (*P* = 0.11)
Improving living condition, infrastructure and service in rural areas	More feasible in Europe and Americas (55% and 45%) and less in Southeast Asia (31%), *P* = 0.031	More feasible for physicians than non-physicians (48% vs 30%), *P* = 0.005	No difference (*P* = 0.62)
Providing safe and supportive environment in rural and remote posts	More feasible in Europe and Americas (48% and 48%) and less in Southeast Asia (29%), *P* = 0.037	More feasible for physicians than non-physicians (45% vs 29%), *P* = 0.013	No difference (*P* = 1.00)
Support career development programmes	No difference (*P* = 0.09)	More feasible for physicians than non-physicians (50% vs 36%), *P* = 0.031	No difference (*P* = 0.83)

Note: Only policy options showing the highest averages for acceptability and feasibility are displayed in this table

Where differences were observed in feasibility, these policy options were often considered as more feasible among respondents in the Americas and Europe than those in other regions. While no differences were identified in acceptability between physicians and non-physicians, feasibility of these policy options were often considered more positively by physicians than non-physicians. For gender, there were no diverging assessments of acceptability or feasibility but for one policy option, *providing safe and supportive environment*, which was considered even more acceptable to the female respondents than their male counterparts.

**Fig. 3:**
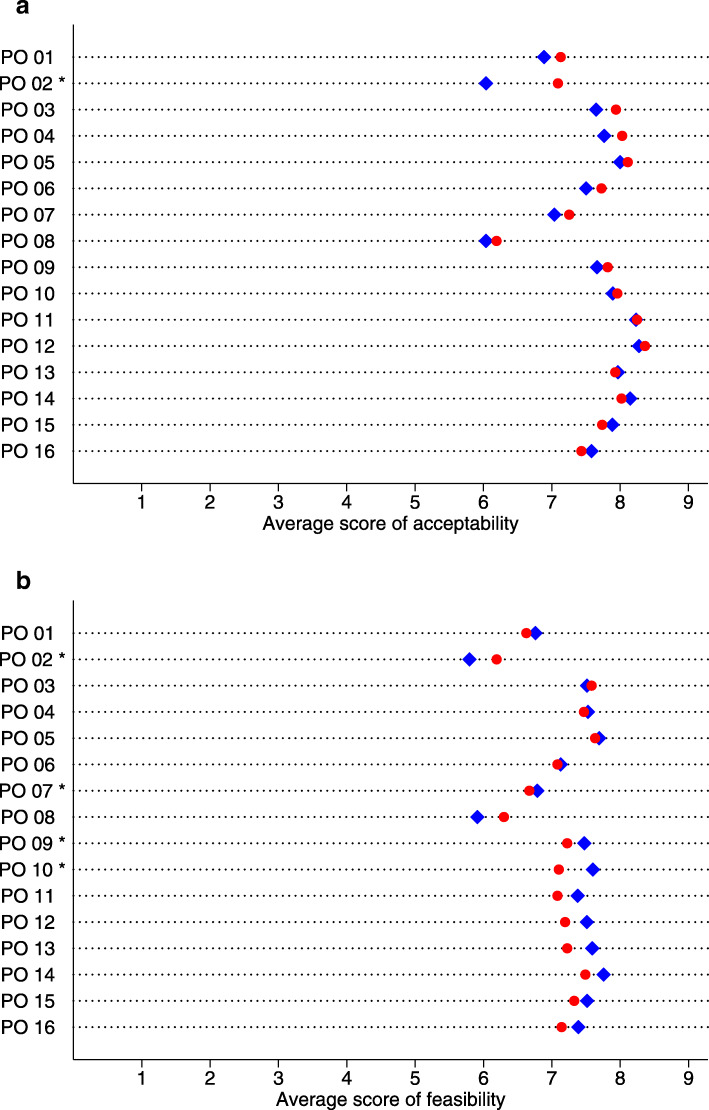
Average score of **a** acceptability and **b** feasibility for each policy option for respondents located in rural or remote area vs others. Legend: blue diamond = respondents in rural or remote areas, red dot = respondents in other locations. *P < 0.05

The acceptability and feasibility ratings of the policy options were also compared between respondents located in rural or remote areas as compared to respondents located elsewhere (Fig. 3). The second policy option on the *location of health profession schools outside major cities* had a lower score for those in rural areas as compared to other respondents for both acceptability (average 6.04 vs 7.09 respectively) and feasibility (average 5.79 vs 6.20 respectively). Policy options 7, 9 and 10, on *production of health workers with appropriate training*, *on educational incentives in exchange of return of service in rural areas* and *financial incentives (monetary and non-monetary*), were considered more feasible by respondents in rural or remote areas compared to others.

### Selected narrative views of respondents on acceptability and feasibility of the policy options

The section presents a narrative summary of relevant comments provided by participants in response to the closed-ended questions relating to the acceptability and feasibility of the outcomes and policy options. Comments were selected if they provided a context-relevant explanation or application of any of the policy options mentioned in the survey. Comments were also considered relevant, if they were directly related to the study objective of understanding stakeholders’ acceptability and feasibility, and the value of the stated outcomes. The selected comments were then grouped according to how they relate to specific or bundled policy options across the four categories: education, regulation, financial incentives and personal and professional support. Most comments were provided by participants from the South and Southeast Asian region and were often about rural experiences in high-income settings. The key views outlined are presented as follows:

- Financial incentives, when effective, are acceptable and feasible for short-term recruitment but are unhelpful for the long-term retention of workers.
- Poor quality of rural primary and secondary training could impede students seeking typically higher academic grades for admission compared to urban students with better training.
- Enforcing compulsory service is generally frowned upon as a means to enhance retention. Instead, employers are advised to create the appropriate environment that stimulates health workers to voluntarily stay in the job. These include access to permanent posts, safe work areas, reasonable accommodation, supported work practice and career development.
- Rural health programme should put trainees at the centre of its planning and orientation. Emphasis should be placed on protecting the pride of rural health programmes and engraining its importance in the psyche of students during the early periods of their training.

Generally, the challenges that were highlighted by participants showed some commonality across settings though proffered solutions were more likely to be unique to the setting of the observer. Considering respondent’s perspectives, these views give additional insight into their personal experiences in implementing or benefiting from the policy options, and the possible implications of this for addressing the recruitment and retention of health workers.

### Need for policy option guidance and barriers to implementation

High percentages of respondents indicated the need for guidance on implementation for each of the four categories of policy options: educational (89%), regulatory (87%), financial incentives (89%), personal and professional support (90%).

**Fig. 4:**
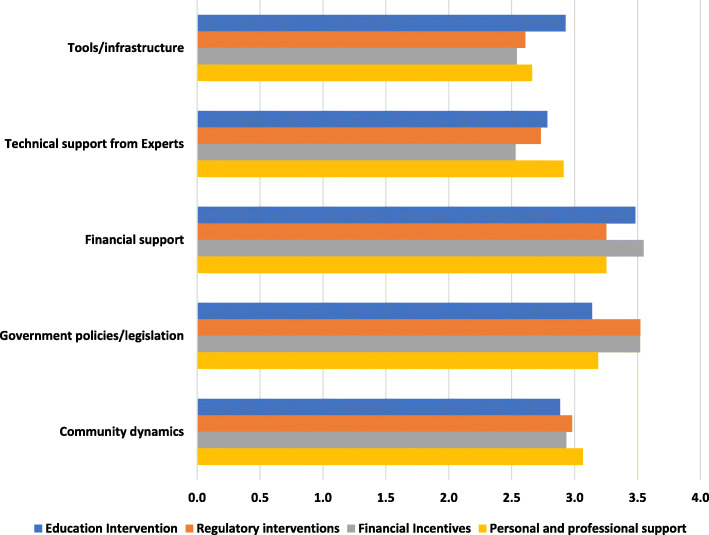
Average ranking of barriers. This figure shows the average ranking of the five barriers on a sliding scale of 1 (least importance) to 5 (highest importance) across the four main areas of policy options for the retention of health workers

Figure 4 summarizes the scores obtained for each of the five potential barriers identified across the four categories of policy options. Across the four categories, the provision of financial support and government policies/legislation were seen as greater barriers compared with the other types of barriers to implementation of the policy options. Concerning the education policy options, tools/infrastructure was ranked as the most important barrier, while still behind financial support and government policies/legislation. There were no observed significant differences by region, or by occupation (comparing physicians vs non-physicians) in the ranking of barriers for the four categories of policy options.

## Discussion

This study assessed the views of stakeholders on the importance of 19 outcomes of interest, and the acceptability and feasibility of 16 policy options aimed at retaining health workers in rural and remote areas. In summary, most of the outcomes were perceived to be critical and the policy options definitely acceptable and definitely feasible by the respondents. The policy options were noted to be generally more acceptable than feasible. Respondents indicated a strong need for guidance on implementation of the policies, particularly for financial support and government incentives. Selected comments reflecting on the closed-ended quantitative findings centred around nuances relating to commonly held views and approaches for enhancing the recruitment and retention of health workers. Highlighted challenges often showed some level of commonality across settings though the solutions proffered were likely to be unique to their setting of interest. An overarching impression, which is consistent with existing evidence [15], is that there is no one-size-fits-all approach, as complexity of expressed challenges may need the combination of several policy options (bundled policies) to help achieve the desired outcome.

In relating some of the qualitative findings of this paper to outcomes in documented literature, similar trends can be observed. For instance, studies have shown that financial incentives—when effective—can indeed promote short-term recruitment but is less effective for the long-term retention of workers [16–19]. Studies have also shown that health workers generally frown upon the enforcement of compulsory service to enhance retention [20, 21]. As an acceptable alternative, policy-makers and employers are advised to create the appropriate environment that stimulate health workers to voluntarily stay in the job [18]. These include but are not limited to access to permanent posts, safe work areas, reasonable accommodation, supported work practice and career development [10, 22, 23]. Finally, there is evidence that protecting the legacy of rural health programmes by engraining its importance early in academic life of students, as well as putting trainees at the centre of rural health programmes, is helpful to improve their retention [24, 25].

### Study limitations and strengths

A key strength of this study is the comprehensive insight it adds to the body of literature in understanding health workers and decision-makers’ preferences and values regarding the WHO retention guideline recommendations. The study also takes a wide-reaching approach and uses multilanguage questionnaires to target participants across all six WHO regions, ensuring key and diverse groups are engaged and included in gathering as comprehensive data as possible.

One limitation of the survey is regarding the distribution of the occupational background of the respondents. About half of the study respondents were physicians, with less representation from nurses, midwives, community health workers and the communities receiving care. To address this, we analysed the data separately for two broad groups (physicians vs. non-physicians) and reported potential discrepancies. Another limitation was our inability to compute a response rate as the survey was distributed through various open communication platforms. Distribution through open platforms did not also allow us to control for self-selection bias. This was however addressed by ensuring that the survey was distributed to a global audience, using regional-level dissemination channels to incorporate as diverse views as possible including stakeholder platforms with direct activities in rural areas.

### Generalizability of findings

The study findings reinforce our understanding and portray a global perspective of stakeholders’ acceptability and feasibility of the policy options outlined for the WHO health worker retention guideline. A recurring theme in literature is the deficiency of adequate remuneration for health workers which usually outranks the other factors that influence recruitment and retention of rural health workers, particularly in the African region. This may explain the high ranking of the *improved motivation* outcome among African respondents, given the prevalence of low remuneration in the region [26, 27]. Insufficient motivation also seems to affect physicians more compared to non-physicians [28, 29]. Respondents representing America and Europe generally provided higher scores for feasibility compared with respondents from other regions. This may be partially explained by the potential complexity of interventions needed, and the differential availability of human and financial resources to address some of the issues which could be more challenging in other regions [30]. For example, though China and Cambodia are geographically close, their socio-economic circumstances differ. In a recent study by Zhu et al., China took a different approach than Cambodia, affording to fund the retention of rural medical doctors, while Cambodia focused its resources on strengthening nurses and medical assistants for its rural practice instead [31].

In addition, female respondents found the policy option on *provision of safe and supportive environment in rural and remote posts* more acceptable than did male respondents to the survey*.* This highlights the importance of gender representation in decision-making, where values may diverge slightly on what is acceptable; this would be particularly relevant if unsafe or unsupportive environments were found to affect females and males differently. Occupations that frequently provide care outside of clinical settings such as midwifery and community health worker roles, are also likely to be most in favour of this policy option [32]. Generally, respondents rated the policy options lower on their feasibility compared to acceptability. One not-surprising finding is that the policy option rated the lowest on feasibility was *Locating health profession schools outside major cities*. This policy option, along with *targeted admissions of students from rural backgrounds*, has been frequently implemented over the years to improve *social accountability—*an outcome rated as critical in our study. Both feasibility and acceptability ratings were higher for *targeted admissions* than they were for *locating schools outside major cities.* While the higher feasibility rating of *targeted admissions* can be expected, it is not clear whether its higher acceptability rating represents a ‘halo effect’, or a real perception. It is important to note also that the implementation of these two policy options often may involve complex processes and strategies including significant political commitment, effective policy coordination and the availability of financial resources which can negatively impact their feasibility if not available [33–35]. On the differences between the acceptability and feasibility ratings of the policy options by those in rural and remote areas versus other locations, it is not clear why the *location of health profession schools outside of major cities* was considered less acceptable and less feasible by respondents located in rural areas. It is however proposed that the higher feasibility accorded to policy options 7, 9 and 10 (*production of health workers with appropriate training, educational incentives in exchange of return of service in rural areas* and *financial incentives* respectively) by respondents located in rural areas could be because these policy options tend to pre-adapt or prepare the recipient to work in rural areas as compared to other policy options that may still afford recipients a wider flexibility to choose non-rural/remote places to work.

### Implications for policy

The value of health workers in rural areas has been ever more highlighted by the impact on rural populations of public health emergencies such as Ebola, and more recently the COVID-19 pandemic [36, 37]. The findings of this study are consistent with stakeholders advocating for an increased investment in decent jobs for all health workers including those in rural and medically underserved areas [38–40]. The findings of this study have been presented along with systematic reviews evidence to support the development of the WHO guideline recommendations aiming to increase health worker recruitment and retention. The general acceptability and feasibility of the policy options and the perceived importance of the outcomes of interest considered in this paper increases the likelihood of its relevance, uptake and ownership by decision-makers and users in countries. To further enhance the successful implementation of the policy options, lessons for all health workers can be drawn from the deliberate orchestration of community engagement mechanisms and strategies that is well established in the community health worker experience. Research has shown that putting these mechanisms in place improves health workers’ motivation as well as their acceptability [41–43]. This could be one of the important links in exploring ways to address perceived discrepancies between how rural-based stakeholders may view certain policy options that they deem less acceptable and feasible compared to their colleagues based in other locations.

### Implications for research

Our study findings add to our understanding of stakeholders’ views on policies to attract, recruit and retain health workers in remote and rural areas. Our findings also present variations in acceptability and feasibility across respondents from the various regions which need to be further explored to expand understanding. Future studies should also ensure that communities receiving care are engaged and that their views are concretely captured as part of the stakeholders’ audience. Standard protocols and methodological approaches to capture the inputs of stakeholders’ acceptability and feasibility of policy options in the development of guideline recommendations are still in the earliest phases of development [44]. This study adds to a small number of evidence pieces detailing relevant methods and approaches necessary for the eventual development of standard methodologies.

Finally, the true achievement of universal health coverage must include the ability of people living in remote and rural areas to access quality and affordable health services without necessarily leaving their communities or undertaking prohibitive travel. Understanding the policy factors that affect the availability of health workers in these often-under-resourced areas is critical to meeting this objective. This paper adds to the pool of knowledge resources that can give insight to policy- and decision-makers in enabling the development and successful implementation of stakeholder-inclusive health worker recruitment and retention policies.

## Supplementary information

**Additional file 1.** Stakeholders’ valuation of retention strategies to increase access to health workers in remote and rural areas

**Additional file 2.** Table S3-Table S4a, b

## Data Availability

Deidentified data can be made available on reasonable request from the corresponding author.

## Abbreviations

HRH: Human resources for health
COVID-19: Corona virus (SARS-CoV-2)
PO: Policy options
UHC: Universal health coverage
WHO: World Health Organization
