# Supplementary material for: Increasing access to health workers in rural and remote areas: what do stakeholders’ value and find feasible and acceptable?
Source: Hum Resour Health. 2020 Oct 16;18:77. doi: 10.1186/s12960-020-00519-2 (PMC7565226; doi:10.1186/s12960-020-00519-2)
Supplement: Supplementary file 1 — Additional file 1. Stakeholders’ valuation of retention strategies to increase access to health workers in remote and rural areas [file 12960_2020_519_MOESM1_ESM.pdf]

Stakeholders' valuation of retention strategies to increase access to health workers in remote and rural areas

Introduction

COMPLETION GUIDE

The World Health Organization (WHO) is updating the2010 policy guidelines on increasing access to health workers through improved retention. In addition to a systematic review of the evidence we are eliciting stakeholder perspectives as to the importance, acceptability, feasibility and use of the various policy options in the 2010 guidelines. If you are a stakeholder your responses to this survey will help inform the guideline revision process.

Note to participants: This survey asks for your perception about the importance, feasibility and acceptability of various interventions which were part of the 2010 guidelines. The information obtained will be analysed and primarily used to support an update of the 2010 guidelines to increase access to health workers in remote and rural areas through improved retention strategies. Responses provided are anonymous and will remain confidential; only aggregate findings will be presented or published by WHO. All questions with an asterisk (*) sign require an answer. Participation in the survey is voluntary and you may withdraw your consent at anytime when undertaking the survey by not clicking the submit button.

We will consider your consent to participate in the study is given when you begin the survey and click on the "NEXT" button at the bottom of the first page. We also consider that you permit us to publish aggregate findings (results will be anonymous untraceable to participants). Completion of the questionnaire should take approximately 20 minutes and you will be able to save your answers as you go along or return to them at a later time. Please complete the ratings from you own perspective, taking into account your specific/local context. You can find descriptions of the outcome measures in the accompanying glossary. After the "Thank you" message is displayed at the end of the survey, do not forget to click on the "SUBMIT" button again.

You may also choose to add explanations to your ratings or offer other comments in the open questions at the end. Should you encounter any questions on which you would like further clarification or guidance, please feel free to contact workforce2030@who.int with your questions. You can find more information regarding the guideline development process at www.who.int/hrh/en

Thank you for completing this survey!

Glossary

--

Terms and Definition

Health workers are all people engaged in actions whose primary intent is to enhance health.

Health labour market is a dynamic system comprising of the economic forces health workers supply and the demand for such workers whose actions are shaped by a country's institutions and regulations.

Integrated care is a concept bringing together inputs, delivery, management and organization of services related to diagnosis, treatment, care, rehabilitation and health promotion. Integration is a means to improve services in relation to access, quality, user satisfaction and efficiency.

Task-shifting (relative to) usual care: Task shifting is a process by which there is delegation of tasks, where appropriate, to less specialized health workers. It presents a viable solution for improving health care coverage by using the already available human resources more efficiently and by quickly increasing capacity while training and retention programmes are expanded.

WHO regions: African region (AFR), Region of the Americas (AMR), Eastern Mediterranean region (EMR), European region (EUR), South-East Asia region (SEAR), Western Pacific region (WPR).

Knowledge: Degree to which the health worker has the theoretical or practical understanding of the function and tasks assigned to him/her

Competencies: Degree to which the health worker has the skills necessary to carry out the tasks assigned to him/her

Motivation: An individual's degree of willingness to exert and maintain effort on assigned tasks

Morale: The mental and emotional condition (as of enthusiasm, confidence, etc.) of an individual health worker with regard to the function or tasks at hand

Satisfaction: Degree to which health workers derive personal satisfaction from serving the community, providing good quality services

Service delivery: Quantity and quality of promotional, preventive, and curative services health workers provide to community members

Responsiveness: The degree to which an individual health worker responds to the needs of an individual client or group within a reasonable time period

Productivity: A health worker's total output per unit of total input

Attrition: The rate at which practicing health workers resign, retire, or abandon their positions over time

Access: Client's physical and social access to essential services delivered by health workers

Coverage: The coverage of selected health services among the population served by health workers

Quality: The quality of services rendered by health workers

Satisfaction: Client's reported degree of satisfaction with the services rendered by health workers

----

* 1. Are you involved in the management or administration of human resources for health in rural and remote areas of your work setting?

- ◯ Yes
- ◯ No

* 2. Is the attraction, recruitment and retention of health workers in rural areas a challenge in your main country of work?

- ◯ Yes
- ◯ No

* 3. Are you based in a rural or remote location?

- ◯ Yes
- ◯ No

* 4. What is your involvement in rural or remote policy or programmes?

- ☐ Not applicable
- ☐ Developing policies /programme decisions
- ☐ Influencing policies/programme decisions

Other (please specify)

Stakeholders’ valuation of retention strategies to increase access to health workers in remote and rural areas

Profile Characteristics

5. Your approximate age

- ○ < 25
- ○ 25 - 29
- ○ 30 - 34
- ○ 35 - 39
- ○ 40 - 44
- ○ 45 - 49
- ○ 50 - 54
- ○ 55 - 59
- ○ 60 - 64
- ○ 65 +

6. Gender

- ○ Female
- ○ Male
- ○ Others

7. Your main organization

- [ ] Non-governmental organization (NGO)
- [ ] Community organization
- [ ] Health professional association
- [ ] Academic institution
- [ ] Governmental organization
- [ ] Academic/ research institution
- [ ] Private for profit organization
- [ ] Labour union
- [ ] Civil society organization
- [ ] Multilateral/International organization
- [ ] Other (please specify)

8. Highest attained educational degree

- ( ) Primary school education
- ( ) Secondary school certificate or diploma
- ( ) Bachelor's degree (e.g. BSc)
- ( ) Medicine degree (MBBS, MD)
- ( ) Master's degree (e.g. MSc, MA)
- ( ) Doctoral degree (PhD or equivalent)
- ( ) None of the above

9. The geographical scope of your work as it pertains to rural and remote health workforce (if applicable)

- [ ] Community/District
- [ ] National (at the country level)
- [ ] Regional (at a level broader than national)
- [ ] International
- [ ] Not applicable

10. Primary region of work

- ◯ Africa
- ◯ South and South East Asia
- ◯ Americas
- ◯ Europe
- ◯ Eastern Mediterranean
- ◯ Western Pacific

Please specify the country(ies) as applicable.

11. Are you a full-time student?

- ◯ Yes
- ◯ No
- ◯ Not applicable

12. Which option(s) best describe your occupation

- [ ] Generalist medical practitioners
- [ ] Specialist medical practitioners
- [ ] Nursing professionals
- [ ] Midwifery professionals
- [ ] Traditional and complementary medicine professionals
- [ ] Paramedical practitioners
- [ ] Dentists
- [ ] Pharmacists
- [ ] Environmental and occupational health and hygiene professionals
- [ ] Physiotherapists
- [ ] Dieticians and nutritionists
- [ ] Community health workers
- [ ] Health services manager
- [ ] Academics/ researchers
- [ ] Policy makers
- [ ] Medical and pharmaceutical technicians
- [ ] Nursing associate professionals
- [ ] Midwifery associate professionals
- [ ] Traditional and complementary medicine associate professionals
- [ ] Health care assistants
- [ ] Home-based personal care workers
- [ ] Other occupation (please specify)

Stakeholders’ valuation of retention strategies to increase access to health workers in remote and rural areas

Assessment of health worker retention outcome indicators

Please rate on the scale of 1-9, how important you think the following outcome indicators are for increasing access to health workers in rural and remote areas.

13. How important are the following outcome indicators for monitoring health worker retention? (NOTE: The first 9 outcome indicators pertain to the workforce while the remaining 10 indicators pertain to the health system)

	Not important (1)	(2)	(3)	(4)	Important (5)	(6)	(7)	(8)	Critical (9)
Improved availability of health workers	○	○	○	○	○	○	○	○	○
Improved competence of health workers	○	○	○	○	○	○	○	○	○
Improved responsiveness of health workers to community needs	○	○	○	○	○	○	○	○	○
Improved productivity of health workers to do tasks more efficiently	○	○	○	○	○	○	○	○	○
Improved public/community recognition of rural/remote health workers	○	○	○	○	○	○	○	○	○
Improved cooperation (between rural and urban health workers)	○	○	○	○	○	○	○	○	○
Improved motivation	○	○	○	○	○	○	○	○	○
Improved personnel development and lifelong learning opportunities	○	○	○	○	○	○	○	○	○
Reduced turnover rate of health workers leaving remote/rural posts	○	○	○	○	○	○	○	○	○
Improved accessibility (coverage of interventions)	○	○	○	○	○	○	○	○	○
Improved quality of care	○	○	○	○	○	○	○	○	○

	Not important (1)	(2)	(3)	(4)	Important (5)	(6)	(7)	(8)	Critical (9)
Improved productivity (of the health system)	◯	◯	◯	◯	◯	◯	◯	◯	◯
Improved social accountability	◯	◯	◯	◯	◯	◯	◯	◯	◯
Improved responsiveness	◯	◯	◯	◯	◯	◯	◯	◯	◯
Improved practice environment (including supportive supervision)	◯	◯	◯	◯	◯	◯	◯	◯	◯
Improved rural recruitment of health workers	◯	◯	◯	◯	◯	◯	◯	◯	◯
Improved rural attractiveness to health workers	◯	◯	◯	◯	◯	◯	◯	◯	◯
Improved workforce skills mix	◯	◯	◯	◯	◯	◯	◯	◯	◯
Improved scope of practice	◯	◯	◯	◯	◯	◯	◯	◯	◯

Stakeholders’ valuation of retention strategies to increase access to health workers in remote and rural areas

Assessment of health worker retention interventions

14. Please rate on a scale of 1-9, how**acceptable** for implementation you think the following health worker retention interventions are:

	Definitely not acceptable (1)	(2)	(3)	(4)	Uncertain whether acceptable or not (5)	(6)	(7)	(8)	Definitely acceptable (9)
1) targeted admissions of students from a rural background into health profession schools									
2) location of health profession schools outside major cities									
3) provide clinical rotations/community experiences in rural areas during pre-service education									
4) revise the curricula of pre-service education to include rural health issues, skills for team-building and supervision, and primary care orientation									
5) continuing education and professional development programmes that meets the needs of rural health workers									
6) enhance the scope of practice of specific cadres of health workers in rural areas									
7) produce different types of health workers with appropriate training and regulation for rural practice									

	Definitely not acceptable (1)	(2)	(3)	(4)	Uncertain whether acceptable or not (5)	(6)	(7)	(8)	Definitely acceptable (9)
8) impose a compulsory service in rural areas in exchange of licensing or other employment benefits									
9) scholarships or other type of financial incentives for education in exchange of return of service in rural or remote areas									
10) provide appropriate financial incentives (monetary or non-monetary)									
11) Improve living conditions for health workers and their families and invest in infrastructure and services in rural areas									
12) provide a safe and supportive working environment for rural and remote posts									
13) implement appropriate outreach support activities									
14) support career development programmes									
15) support the development of professional networks									
16) adopt public recognition measures									

15. Please rate on a scale of 1-9, how**feasible** for implementation you think the following health worker retention interventions are:

	Definitely not feasible (1)	(2)	(3)	(4)	Uncertain whether feasible or not (5)	(6)	(7)	(8)	Definitely feasible (9)
1) targeted admissions of students from a rural background into health profession schools									

	Definitely not feasible (1)	(2)	(3)	(4)	Uncertain whether feasible or not (5)	(6)	(7)	(8)	Definitely feasible (9)
2) location of health profession schools outside major cities	○	○	○	○	○	○	○	○	○
3) provide clinical rotations/community experiences in rural areas during pre-service education	○	○	○	○	○	○	○	○	○
4) revise the curricula of pre-service education to include rural health issues, skills for team-building and supervision, and primary care orientation	○	○	○	○	○	○	○	○	○
5) continuing education and professional development programmes that meets the needs of rural health workers	○	○	○	○	○	○	○	○	○
6) enhance the scope of practice of specific cadres of health workers in rural areas	○	○	○	○	○	○	○	○	○
7) produce different types of health workers with appropriate training and regulation for rural practice	○	○	○	○	○	○	○	○	○
8) impose a compulsory service in rural areas in exchange of licensing or other employment benefits	○	○	○	○	○	○	○	○	○
9) scholarships or other type of financial incentives for education in exchange of return of service in rural or remote areas	○	○	○	○	○	○	○	○	○
10) provide appropriate financial incentives (monetary or non-monetary)	○	○	○	○	○	○	○	○	○
11) Improve living conditions for health workers and their families and invest in infrastructure and services in rural areas	○	○	○	○	○	○	○	○	○

	Definitely not feasible (1)	(2)	(3)	(4)	Uncertain whether feasible or not (5)	(6)	(7)	(8)	Definitely feasible (9)
12) provide a safe and supportive working environment for rural and remote posts	○	○	○	○	○	○	○	○	○
13) implement appropriate outreach support activities	○	○	○	○	○	○	○	○	○
14) support career development programmes	○	○	○	○	○	○	○	○	○
15) support the development of professional networks	○	○	○	○	○	○	○	○	○
16) adopt public recognition measures	○	○	○	○	○	○	○	○	○

16. Where you would like to clarify, please provide an explanation for chosen answers to any of the questions above. (Please indicate the specific number of the question before providing your answer)

17. Other comments: (250 characters max.)

Stakeholders' valuation of retention strategies to increase access to health workers in remote and rural areas

Guidance on implementation

18. Specific guidance (such as a checklist or 'how to' guide) is needed to help implementation of **education interventions** for rural health workers:

- Yes
- No

19. Specific guidance (such as a checklist or 'how to' guide) is needed to help implementation of **regulatory interventions** for rural health workers:

- Yes
- No

20. Specific guidance (such as a checklist or 'how to' guide) is needed to help implementation of **financial incentives** for rural health workers:

- Yes
- No

21. Specific guidance (such as a checklist or 'how to' guide) is needed to help implementation of **personal and professional support** for rural health workers:

- Yes
- No

22. Please rank in order of importance (from lowest (1) to highest (5) the following barriers to implementing **educational interventions** for rural health workers

- Tools/Infrastructure
- Technical support from experts
- Financial support
- Government policies/legislation
- Community dynamics

23. Please rank in order of importance (from lowest (1) to highest (5) the following barriers to implementing **regulatory interventions** for rural health workers

- Tools/Infrastructure
- Technical support from experts
- Financial support
- Government policies/legislation
- Community dynamics

24. Please rank in order of importance (from lowest (1) to highest (5) the following barriers to implementing **financial incentives** for rural health workers

- Tools/Infrastructure
- Technical support from experts
- Financial support
- Government policies/legislation
- Community dynamics

25. Please rank in order of importance (from lowest (1) to highest (5) the following barriers to implementing **personal and professional support** for rural health workers

- Tools/Infrastructure
- Technical support from experts
- Financial support
- Government policies/legislation
- Community dynamics
