# Supplementary material for: Increasing access to health workers in rural and remote areas: what do stakeholders’ value and find feasible and acceptable?
Source: Hum Resour Health. 2020 Oct 16;18:77. doi: 10.1186/s12960-020-00519-2 (PMC7565226; doi:10.1186/s12960-020-00519-2)
Supplement: Supplementary file 2 — Additional file 2. Table S3-Table S4a, b [file 12960_2020_519_MOESM2_ESM.pdf]

Table 3: Values attached to outcome of interest; answers provided on a 9-point Likert scale

Outcome	*N*	*Mean*	*Median*	*Interquartile range*	*% with a '9' rating*
Workforce performance					
**Improved availability of health workers**	**284**	**7.5**	**8**	**[6-9]**	**45%**
Improved competence of health workers	284	7.3	8	[6-9]	37%
Improved responsiveness of health workers to community needs	285	7.2	8	[6-9]	31%
Improved productivity of health workers to do tasks more efficiently	285	6.8	7	[5-8]	22%
Improved public/community recognition of rural/remote health workers	285	7.3	8	[6-9]	36%
Improved cooperation (between rural and urban health workers)	285	7.0	7	[5-9]	31%
**Improved motivation**	**281**	**7.4**	**8**	**[6-9]**	**42%**
Improved personnel development and lifelong learning opportunities	284	7.4	8	[6-9]	36%
Reduced turnover rate of health workers leaving remote/rural posts	283	7.3	8	[6-9]	39%
Health systems					
Improved accessibility (coverage of interventions)	284	7.3	8	[6-9]	36%
Improved quality of care	281	7.3	8	[6-9]	33%
Improved productivity (of the health system)	282	7.1	7	[6-9]	31%
Improved social accountability	283	7.0	7	[5-9]	27%
Improved responsiveness	283	7.0	7	[5-8]	25%
Improved practice environment (including supportive supervision)	283	7.4	8	[6-9]	34%
**Improved rural recruitment of health workers**	**285**	**7.3**	**8**	**[6-9]**	**40%**
**Improved rural attractiveness to health workers**	**284**	**7.8**	**9**	**[7-9]**	**52%**
Improved workforce skills mix	284	7.0	7	[5-9]	26%
Improved scope of practice	284	6.7	7	[5-8]	20%

Table 4a: Stakeholders' perception of the acceptability of the guidelines policy options; answers provided on a 9-point Likert scale

	Acceptability				
	N	*Mean*	*Median*	*Inter quartile range*	*% with a '9' rating*
Targeted admissions of students from a rural background into health profession schools	258	7	7	[5-9]	37%
Location of health profession schools outside major cities	257	6.4	7	[5-9]	30%
Provide clinical rotations/community experiences in rural areas during pre-service education	256	7.8	9	[7-9]	51%
Revise the curricula of pre-service education to include rural health issues, skills for team-building and supervision, and primary care orientation	258	7.9	8.5	[7-9]	50%
Continuing education and professional development programs that meets the needs of rural health workers	257	8	9	[7-9]	54%
Enhance the scope of practice of specific cadres of health workers in rural areas	258	7.6	8	[7-9]	38%
Produce different types of health workers with appropriate training and regulation for rural practice	256	7.1	8	[6-9]	32%
Impose a compulsory service in rural areas in exchange of licensing or other employment benefits	257	6.1	6	[5-8]	23%
Scholarships or other type of financial incentives for education in exchange of return of service in rural or remote areas	256	7.7	8	[7-9]	50%
Provide appropriate financial incentives (monetary or non-monetary)	258	7.9	9	[7-9]	52%
**Improve living conditions for health workers and their families and invest in infrastructure and services in rural areas**	**256**	**8.2**	**9**	**[8-9]**	**64%**
**Provide a safe and supportive working environment for rural and remote posts**	**258**	**8.3**	**9**	**[8-9]**	**66%**
Implement appropriate outreach support activities	257	8	9	[7-9]	53%
Support career development programs	255	8.1	9	[7-9]	56%
Support the development of professional networks	257	7.8	8	[7-9]	48%
Adopt public recognition measures	254	7.5	8	[6-9]	44%

Table 4b: Stakeholders' perception of the feasibility of the guidelines policy options; answers provided on a 9-point Likert scale

	Feasibility				
	N	***Mean***	***Median***	***Inter quartile range***	***% with a '9' rating***
Targeted admissions of students from a rural background into health profession schools	257	6.7	7	[5-9]	30%
Location of health profession schools outside major cities	255	5.9	6	[5-8]	20%
**Provide clinical rotations/community experiences in rural areas during pre-service education**	**252**	**7.5**	**8**	**[7-9]**	**44%**
**Revise the curricula of pre-service education to include rural health issues, skills for team-building and supervision, and primary care orientation**	**253**	**7.5**	**8**	**[6-9]**	**43%**
**Continuing education and professional development programs that meets the needs of rural health workers**	**254**	**7.7**	**8**	**[7-9]**	**43%**
Enhance the scope of practice of specific cadres of health workers in rural areas	254	7.1	7	[6-9]	30%
Produce different types of health workers with appropriate training and regulation for rural practice	252	6.7	7	[5.5-9]	27%
Impose a compulsory service in rural areas in exchange of licensing or other employment benefits	253	6.1	6	[5-8]	24%
Scholarships or other type of financial incentives for education in exchange of return of service in rural or remote areas	254	7.4	8	[6-9]	39%
**Provide appropriate financial incentives (monetary or non-monetary)**	**253**	**7.4**	**8**	**[6-9]**	**42%**
Improve living conditions for health workers and their families and invest in infrastructure and services in rural areas	254	7.3	8	[6-9]	40%
Provide a safe and supportive working environment for rural and remote posts	254	7.4	8	[6-9]	38%
Implement appropriate outreach support activities	252	7.4	8	[6-9]	37%
**Support career development programs**	**254**	**7.7**	**8**	**[7-9]**	**44%**
**Support the development of professional networks**	**253**	**7.4**	**8**	**[7-9]**	**41%**
Adopt public recognition measures	253	7.3	8	[6-9]	39%
